# Perception of Cyberbullying in Adolescence: A Brief Evaluation Among Italian Students

**DOI:** 10.3389/fpsyg.2020.607225

**Published:** 2020-11-25

**Authors:** Valeria Saladino, Stefano Eleuteri, Valeria Verrastro, Filippo Petruccelli

**Affiliations:** ^1^Department of Human Sciences, Society and Health, University of Cassino and Southern Lazio of Cassino, Cassino, Italy; ^2^Department of Psychology, Sapienza University of Rome, Rome, Italy; ^3^Department of Medical and Surgical Sciences, University “Magna Graecia” of Catanzaro, Catanzaro, Italy; ^4^Faculty of Economics, Mercatorum University, Rome, Italy

**Keywords:** adolescence, cyberbullying, digital devices, intervention, psychological disease, suicide

## Abstract

Cyberbullying is associated with the expansion of digital devices and the Internet. In Italy and other European and non-European countries, the phenomenon is growing. Young people who suffer from cyberbullying develop psychopathological symptoms of anxiety, depression, and social phobia that can lead to extreme acts, such as suicide. The pressure, the sense of isolation, and helplessness experienced by cyber-victims also affect their family and the school context. Cyberbullying is acted through digital tools, it is often anonymous, and aims to destroy and psychologically humiliate the victim. There are various forms of cyberbullying that involve different reactions and consequences. However, few studies have focused on adolescents’ perception of cyberbullying. Youths often engage in aggressive behaviors, ignoring the feelings and reactions of the victims. Based on these considerations, our article aims to provide a general overview of the spread of the phenomenon and to understand the various types of cyberbullying and its consequences on victims. We will also illustrate a brief evaluation conducted in Italian schools investigating the perception of cyberbullying in a sample of 600 Italian adolescents (11–14 years old). Our work aims to investigate the cognition and the personal perception of youths about cyberbullying and its consequences and to promote educational interventions within and outside the context of school.

## Introduction

Recent technological developments have led to the progressive evolution of the human relationship concept. Voluntary access to social networks and online communities implies immediate proximity mediated by the web (Pratono, 2018; Auriemma et al., 2020). This evolution in human relations happened very quickly and mainly involved younger generations (Eleuteri et al., 2017). Parents, teachers, and adults are not always aware of the functioning, the rules, or the risks of the web and very often witness acts of violence and abuse among peers, which can end in tragic and extreme consequences.

Cyberbullying or electronic bullying is one of the well-known risks of this technological evolution and consists of voluntary and repeated actions against one or more individuals, through the use of computers and electronic devices (Aboujaoude et al., 2015). Cyberbullying is characterized by the following elements: *voluntary act*, the behavior is intentional and not accidental; *repeated act*, the behavior is repeated over time and not reduced to a single event; *perception of damage by the victim*, the victim suffers the damage inflicted; *use of electronic devices*, cyberbullying is carried out through the use of computers, cell phones, and other electronic means (Ferrara et al., 2018). Cyberbullying, like conventional bullying, is based on an asymmetrical power relationship exercised by the cyber-bully toward the victim (Durak and Saritepeci, 2020). The principal characteristic of cyberbullying is the anonymity guaranteed by the web, which provokes a perception of weakness and loneliness in the victims (Cao et al., 2020). Isolation from the peer group, lower self-esteem, and social anxiety are the most common consequences for cyber-victims. A cyber-bully might circulate images or send offensive messages or e-mails against a person or a group of people in order to deconstruct the perception of security of the victims, humiliating and isolating them. The persecutor who engages in online violence has a lower perception of responsibility for the suffering caused by one’s behavior, underestimating the seriousness of the consequences for the victim, who feels not able to defend from virtual harassment.

In Italy, cyberbullying is one of the main discussed topics among middle and high school students. According to the *Italian Observatory for Family, Social Policy, and Security*, in 2017, 7% of adolescents aged between 11 and 13 years old (specially females) had been the victim of cyberbullying once or more per month (Intrieri and Corraro, 2019). In European and non-European Countries, the rates of cyberbullying vary substantially between studies, with figures ranging from 6.5 to 72% for cyber victimization (Athanasiou et al., 2018; Baldry et al., 2018). For instance, a survey conducted in the United States in 2017 indicates that 33.8% of adolescents have reported that they were the victim of cyberbullying in their lifetime. In Brazil, in a 2017 survey on the use of the Internet by youths aged 9–17, 22% reported being offended online, while 39% reported seeing someone being discriminated against. In China, research published between 2013 and 2018 indicates that cybervictimization ranges from between 14 and 57% and cyber aggression ranges from between 3 and 35% (United Nations Educational, Scientific, and Cultural Organization, 2017). The Global school-based student health survey (GSHS), which collects data from people aged 11, 13, and 15, suggests that the incidence of different types of violence and school bullying seems to change with age (Pontifical Scholas Occurrentes Foundation, 2019). Data from three national surveys in the United States (United Nations Educational, Scientific, and Cultural Organization, 2017) show that the most common forms of bullying, including verbal abuse, theft, threats, defamation, and social exclusion, tend to decrease with age, and bullying is reduced by nearly 50% between ages 14 and 18, while cyberbullying declines at a lower rate, from 17 to 13%. Other research shows that the incidence of bullying in the form of physical aggression is more diffused among primary school students, while cyberbullying occurs more among middle and high school students, increasing in the latter group. In Italy (ISTAT, 2015) data published in 2015 shows that, among mobile and/or Internet users, aged 11–17, 5.9% of them report being bullied via messages email, chat, or social networks. In 2016, police data reported 235 cases of cyberbullying (Commissariato PS, 2016). According to the ISTAT in 2018, 85.8% of children between 11 and 17 years old use mobile phones every day and 72% of children in the same age group use the Internet daily. Mostly girls aged between 14 and 17 years old uses mobile phones (73.2%) and social networks (84.9%). As a consequence, female teenagers are more likely to be the victim of cyberbullying 7.1% of girls suffer from constant harassment online, compared to 4.6% of boys. There is also a greater risk for youths aged between 11 and 13 years old who have been victims of bullying via mobile phones or the Internet once or more times a month (7%) compared to the experiences of youths aged 14–17 (5.2%) (ISTAT, 2019).

Given the serious diffusion of the phenomenon, in Italy cyberbullying is recognized as a crime by law 71/17 (Senato della Repubblica, 2017). The legislation provides a specific legal definition of cyberbullying and requires schools to educate children to use the Internet and to supervise the problem. According to the law cyberbullying is “any form of pressure, aggression, harassment, blackmail, insult, denigration, defamation, identity theft against minors, carried out electronically.” The Ministry of Education has also implemented guidelines for the prevention of cyberbullying (Sorrentino et al., 2018) to counteract this high rate of incidence.

## Types of Cyberbullying and Risks for Health in Adolescence

Cyberbullying is a very complex phenomenon, consisting of different types of behavior, which are outlined here.

*Flaming:* involves sending violent and vulgar messages by an electronic device to provoke verbal conflicts between two or more people within the network. The victim is not always present (Qodir et al., 2019). Flaming occurs during a chat conversation or interactive video games. Mostly flaming is diffused within interactive games since, many times, the victims are beginners targeted by more experienced players (Sung Je et al., 2019).

*Harassment*: includes sending repeated and offensive messages to a specific person, causing strong psychological and emotional distress. Harassment occurs through email, messages, forums, chats, and discussion groups (Wells et al., 2019). As in traditional bullying, the victim is always in a “one down” position and suffers passively from aggression (Choi and Kruis, 2020; Moneva et al., 2020).

*Cyberstalking:* harassment, violence, threats, and persecutions online, toward a person to isolate and frighten them. The cyberstalker has control of their victims and virtually follows them. The persecutor can systematically try to contact the victim, sending offensive and intrusive messages (Reyns and Fissel, 2020). The cyberstalker is protected by anonymity and often uses fake profiles. This contributes to increasing the level of disinhibition (disinhibition effect) and aggression toward the victim (Algeri et al., 2019; Dhillon and Smith, 2019; Saladino et al., 2020).

*Denigration:* involves the distribution of false or derogatory messages toward victims, to damage their reputation or friendships. The persecutor often sends or publishes images, photographs, or videos relating to the victim (Sangwan and Bhatia, 2020). The spectators who receive these types of messages become passive (when they just watch) or active (when they share material with other friends).

*Impersonation*: occurs when a cyber-bully has access to the personal account information of the victim (name and password) and impersonates the target online, uploading negative information that damages the target’s social relationships. This can take place on the target’s social network account, blog, or other forms of online platforms. In more extreme cases, the cyber-bully changes the victim’s password by preventing access to his/her account. The cyber-bully, using this method of aggression, endangers the victim. Furthermore, adolescents often share their passwords to demonstrate their “true friendship” and this indirectly contributes to impersonation (Koch, 2017).

*Tricky or outing:* The cyber-bully becomes a friend of the victim, leads him/her to share private and intimate information. After that stage, the cyber-bully spreads or utilizes the information to threaten the victim (Intrieri and Corraro, 2019).

*Exclusion:* is when the cyber-bully decides to exclude another user from his/her group of friends, or a particular chat or interactive game (password-protected environments). This type of behavior is called “banning.” Exclusion from the group of friends is perceived as a severe type of punishment and can lead to reduced popularity among the peer group and therefore also of the perceived “power” (Willard, 2007; Menesini et al., 2012; Ashktorab and Vitak, 2016).

*Happy slapping:* is associated with traditional bullying, and consists of a video recorded during which the victim is filmed undergoing various forms of violence to humiliate them. The recordings can then be published online and viewed by other internet users (Željka et al., 2019).

According to a survey conducted by UNICEF (2020), victims of cyberbullying are more likely to incur alcohol and drug use and skip school than other students. They are also more likely to receive poor grades and have self-esteem and health issues. Young people who have been victims of cyberbullying are often reluctant to confide in adults. Cyberbullying could present symptoms similar to post-traumatic stress disorder, which can lead to suicide (Liu et al., 2020). Experiences of bullying and cyberbullying are often associated with the development of depression, social anxiety (Chu et al., 2018; Wang et al., 2019), and mental health problems (Fahy et al., 2016). Furthermore, cyber-victims have a worse quality of life, especially in terms of their psychological well-being and in the school environment (González-Cabrera et al., 2018). In Italy, there have been prominent media cases such as that of Carolina Picchio or Andrea Spezzacatena (Manes, 2013; Intrieri and Corraro, 2019). Carolina Picchio committed suicide after that her angry ex-boyfriend hurled insults and broadcasted videos in which the girl appeared in intimate attitudes. After weeks of taunts and verbal harassment, the 14-year-old girl threw herself from the window of her home (Polidori, 2018). Andrea Spezzacatena has a different story, which began with his mother who did not separate her son’s jeans from colored garments, and some were were dyed pink. Andrea, amused by the situation, wore them to school but the classmates perceived Andrea to be strange and started to use homophobic appellatives. They created a profile on a well-known social network entitled: “The boy with the pink pants.” The insults and jokes were repeated and followed, ever heavier, until the day when one of the classmates painted offensive homophobic writing on the wall. As a result of this behavior, Andrea became desperate, grabbed a scarf, tied it around his neck until he suffocated, killing himself (Guerriero, 2020).

These examples suggest the need to recognize symptoms of distress in adolescents who might hide the strong feelings of loneliness and sadness associated with the victimization caused by this cyberbullying.

## The Present Study: Adolescents’ Perception of Cyberbullying and Future Directions

Our study explores the personal perception of cyberbullying by youths, taking into account the following types of cyberbullying: Harassment; Tricky or Outing; Denigration; Exclusion; Impersonation, with the aim to: (a) evaluate the general perception of cyberbullying motivation; (b) assess the identification process with the victim and the gender attribution of the cyberbullying acts; (c) evaluate the participants’ tendency to perform cyberbullying, and (d) identify possible gender differences in the personal perception of cyberbullying. The sample examined Italian adolescents.

### Methods and Measure

We recruited 600 participants, divided into 300 males (mean age 12.45; SD 0.900; age range: 11–14) and 300 females (mean age 12,43; SD 0.857; age range: 11–14), recruited from five lower secondary schools in the Central South area of Italy.

The researchers explained the aims and scope of the research to children and their parents and obtained the informed consent for children, signed by the parents, and authorization from the School Director. The participants completed a questionnaire on the perception of cyberbullying, structured by the Department of Human, Social and Health Sciences of the University of Cassino and Southern Lazio. The questionnaire lasted 15 min and proposed five cyberbullying scenarios: (a) Harassment; (b) Tricky or Outing; (c) Denigration; (d) Exclusion; and (e) Impersonation. For each scenario, the participant was asked to answer five multiple-choice questions: (1) cyber-bully motivation; (2) the cyber-victim’s reaction; (3) the cyber-victim’s emotions; (4) the gender attribution of the cyberbully; and (5) their personal propensity to perform acts of cyberbullying. The study was approved by the International Review Board of the University of Cassino and Southern Lazio. Data were analyzed using Statistical Package for Social Sciences (Version 25.0, SPSS Inc., Armonk) (IBM Corp. Released, 2017). Descriptive analysis was used to evaluate the perception of cyberbullying for each scenario proposed. Furthermore, a chi-square test was used to evaluate gender differences in the attribution of cyberbullying acts (Wright, 1992).

All *p*-values were two-tailed, and the level of statistical significance was set at *p* < 0.05 for all tests.

### Results

We undertook a descriptive analysis of the first question and the motivation to cyber-bully shows a strong tendency in participants who attribute the fault to the victim. Indeed, participants gave the same answer for all scenarios, affirming that the cyber-victim suffers from cyberbullying because “he/she is strange and different from others.”

Regarding the second question about the reaction of the cyber-victim in a scenario where they experience Harassment, 30% of the sample thought that the cyber-victim did not react out of fear in the Tricky or Outing, Denigration, and Impersonation scenarios, with results of 32.8, 36.2, and 45.7%, respectively. The participants thought that the cyber-victim should confide in someone (friends, parents, and/or teachers). Only the Exclusion scenario is connected to a different perception of the cyber-victim’ reaction: indeed, 25.2% of the participants affirm that the cyber-victim tries to contact the cyber-bully to ask for an explanation. This reaction might be associated with a lower fear toward the bully, due to the kind of violence or extremity of their behavior. Indeed, exclusion is a type of indirect violence that isolates the victim from others. Mostly, the exclusion could solicit a need for clarity and understanding among the cyber-victims, which can lead them to ask for an explanation.

In response to the third question, on the cyber-victim’s emotions, the answers of participants are coherent with the contents of each scenario, demonstrating the participant’s high capacity for understanding and perceive the feelings of others in different situations of cyberbullying. For instance, in the *Denigration* (20.9%) and *Impersonation* (22.7%) scenarios, participants thought that the cyber-victim felt rage; while in the *Tricky or Outing* scenario, they attribute the feeling of shame to the cyber-victim (28.2%). In the *Harassment* scenario, the most frequently reported emotion was fear (44.8%) while in the *Exclusion* scenario, it was sadness.

An interesting aspect arose concerning question number four, on the gender attribution of the cyberbully. Overall, the participants perceived males as more likely to commit cyberbullying than females. However, the chi-square analysis showed that the percentage of females who engage in acts of cyberbullying to be more than males in the scenarios n. 1 Harassment, 3 Denigration, and 5 Impersonation and was significantly higher than the male group. This perception seems to change in scenario n.2, Tricky or Outing, and in n. 4 on Exclusion, that do not involve aggression or threatening acts. Table 1 reports the percentages of male and female participants in the attribution of cyberbullying, divided for each scenario; the chi-square and the *p-value*.

**TABLE 1:** Percentage of the two groups of participants (male and female), in the attribution of cyberbullying, divided for each scenario.

Same gender attribution of cyberbullying for each scenario	Gender		Chi square (χ^2^)	*p-Value*
	Male	Female		
Scenario n.1 Harassment	91.6%	60%*	82.07	<0.01
Scenario n. 2 Tricky or Outing	73.3%	69.7%	0.98	0.31
Scenario n. 3 Denigration	81.6%	66.2%*	18.54	<0.01
Scenario n. 4 Exclusion	76%	69.3%	3.35	0.06
Scenario n. 5 Impersonation	81.6%	60%*	34.08	<0.01

*Note:* Pearson’s χ^2^ *p < 0.01 value.

Finally, concerning the final fifth question on participants’ tendency to perform cyberbullying, the participants affirmed that they do not commit cyberbullying, showing a strong perception about the consequences of cyberbullying.

Data from the descriptive analysis of each scenario are reported below, showing the percentage of the distribution of the answers among the sample recruited (Table 2).

**TABLE 2:** Perception of cyberbullying for each scenario in the total sample.

Question	Answer	Percentage
**Scenario n.1 Harassment**		
One of your schoolmates is receiving offensive messages and insults:		
*According to you why?*	Because they are strange and different from others	59.5%
*According to you how the cyber-victim reacts?*	The cyber-victim does not react out of fear	30%
*According to you what kind of emotion the cyber-victim feels?*	Fear	44.8%
*According to you the cyber-bully is a male or a female?*	Male	65.8%
*If you had a reason, would you send offensive messages and insults?*	No	69.7%
**Scenario n. 2 Tricky or Outing**		
Someone has gained the trust of one of your schoolmates for the purpose of spreading their personal information online:		
*According to you why?*	Because they are strange and different from others	58.8%
*According to you how the cyber-victim reacts?*	The cyber-victim talks with someone (friends, parents, teachers)	32.8%
*According to you what kind of emotion the cyber-victim feels?*	Shame	28.2%
*According to you the cyber-bully is a male or a female?*	Male	51.8%
*If you had a reason, would you become friend of a person with the goal to spread his/her personal information online?*	No	76.2%
**Scenario n. 3 Denigration**		
Someone is spreading online false and humiliating information about one of your schoolmates:		
*According to you why?*	Because they are strange and different from others	59%
*According to you how the cyber-victim reacts?*	The cyber-victim talks with someone (friends, parents, teachers)	36.2%
*According to you what kind of emotion the cyber-victim feels?*	Rage	20.9%
*According to you the cyber-bully is a male or a female?*	Male	57.8%
*If you had a reason, would you spread online false and humiliating information about someone?*	No	73.9%
**Scenario n. 4 Exclusion**		
Someone is excluding from chat group online one of your schoolmates:		
*According to you why?*	Because they are strange and different from others	55.2%
*According to you how the cyber-victim reacts?*	The cyber-victim tries to contact the cyber-bully to ask for an explanation.	25.2%
*According to you what kind of emotion the cyber-victim feels?*	Sadness	30.5%
*According to you the cyber-bully is a male or a female?*	Male	53.3%
*If you had a reason, would you delete an account or exclude from chat group online someone?*	No	63.4%
**Scenario n. 5 Impersonation**		
Someone has appropriated the account of one of your schoolmates and is posting photos and messages in his/her name.		
*According to you why?*	Because he/she is strange and different from others	54.7%
*According to you how the cyber-victim reacts?*	The cyber-victim talks with someone (friends, parents, teachers)	45.7%
*According to you what kind of emotion the cyber-victim feels?*	Rage	22.7%
*According to you the cyber-bully is a male or a female?*	Male	60.7%
*If you had a reason, would you take over someone else’s account and post photos and messages in his/her name?*	No	76.5%

## Discussion

This research represents the scenario of a specific sample; a preliminary investigation that aimed to generate a hypotheses that will be tested in subsequent studies *ad hoc* designs. The results are not generalizable and could be expanded in the future with a historical or longitudinal investigation. Data suggest that comparative future studies could look at other schools, such as those in the North of Italy, also considering the socio-economic level and the family structure of the participants. In the last few years, there has been a surge of research on cyberbullying, investigating the diffusion and the consequences of the phenomenon. Data found a strong relation between cyber-victimization, anxiety, and depression (Chu et al., 2018; Wang et al., 2019). Furthermore, in European and non-European countries, the rate of suicide is growing, especially among adolescents (Athanasiou et al., 2018). The personal perception of cyberbullying is an important and often undervalued element. This aspect can help in understanding the motivation of cyberbullying, the identification with the cyber-victim, and the tendency to become a cyber-bully. We investigated the topic of adolescents’ perception of cyberbullying taking into account the following types: harassment, tricky or outing, denigration, exclusion, impersonation.

Regarding the first objective to evaluate the general perception of cyberbullying motivation, data showed an attribution of fault to the cyber-victim, emphasizing the cognitive mechanism of dissociation between actions and consequences which can lead to a projected displacement of responsibility of the spectator toward the cyber-bully. Participants affirm that the cyber-victim is targeted due to their personal characteristics and attitudes (they are strange and different from others). Concerning the second objective to assess the identification process with the victim and the gender attribution of cyberbullying acts, participants had a coherent identification with the feelings and possible reactions of the cyber-victim, as shown in the results. In the global analysis of the sample, the majority of the participants attributed acts of cyberbullying to males, showing a high tendency to perceive men as more aggressive than women. With regard to the third objective to evaluate the participants’ tendency to perform cyberbullying, adolescents affirmed that they would not commit any acts of cyberbullying. Finally, concerning the last objective to evaluate the gender differences in the attribution of cyberbullying, there was a significant difference between boys and girls in the attribution of cyberbullying to their identified gender for the scenario n. 1, Harassment; n. 3, Denigration; and 5, Impersonation, all scenarios characterized by aggressive and threatening behavior. Indeed, a high percentage of both groups’ participants attributed cyberbullying to males in all scenarios. Therefore, it might be assumed that there was a perception of the male gender as more aggressive and violent than female. According to these results, men perceive themselves to be more likely to commit cyberbullying, especially aggressive, violent and threatening types; while females perceive themselves as less likely to cyberbully, except for in scenarios n. 2 and n. 4. The Tricky or Outing scenario might be interpreted by the participants as joking with other people and the Exclusion scenario as a form of isolating someone; none of them involves the use of aggression, verbal attacks, threats, or explicit humiliation, acts often attributed to male gender. The participants could underestimate the psychological effects that all cyberbullying types have on victims.

The results of this study can be used to make recommendations to institutions, such as schools, to prevent cyberbullying and its consequences for adolescents. In recent years, school operators have re-evaluated educational and training aspects in addition to the traditional, educational, and cultural ones. Episodes of bullying, oppression, and aggression are often confused and labeled as a generic manifestation of “rudeness.” To reduce cyberbullying, it is important to avoid attribution of blame and focus more on prevention. The study and the possibility to understand the phenomenon should guarantee its inclusion in the education of young people. According to these considerations, we suggest the following approaches.

*Prevention.* Promoting collaboration between family, school, and territory to counter the spread of cyberbullying and to provide socio-educational tools for parents, teachers, and students; implementing communication and confidence among youths and adults and developing new space to train parents and teachers in recognizing cyberbullying.

*Intervention.* Supporting psychologically the cyber-victims, developing face to face and online spaces to sensitize students with aggressive tendencies to the consequences and responsibility of their actions. Interventions could improve the development of communication, socialization, and interpersonal skills among students. An example of interventions focused on these principles is that of the so-called restorative schools (Gregory et al., 2016). This concept derives from restorative justice, which can be applied to the school system to manage conflicts in adolescence.

Only five different types of cyberbullying have been considered in our research, in particular, those most related to educational institutions, which are widespread among very young populations. However, phenomena such as happy slapping, flaming, and cyberstalking are also spreading, with worrying consequences. One of our future goals will be to promote research that also takes into account these types of cyberbullying, expanding this sample, and producing data to promote the psychological well-being in young adults and to support the families of both cyber-victim and cyber-bully.

## Data Availability Statement

The raw data supporting the conclusions of this article will be made available by the authors, without undue reservation.

## Ethics Statement

The studies involving human participants were reviewed and approved by the Institutional Review Board, University of Cassino. Written informed consent to participate in this study was provided by the participants’ legal guardian/next of kin.

## Author Contributions

VS, SE, and VV conceptualized the contribution. VS wrote the manuscript. SE and FP reviewed the manuscript. VS provided the critical revision processes as PI. All authors approved the submission of the manuscript.

## Conflict of Interest

The authors declare that the research was conducted in the absence of any commercial or financial relationships that could be construed as a potential conflict of interest.
